# Influence of Equatorial CH⋅⋅⋅O Interactions on Secondary Kinetic Isotope Effects for Methyl Transfer

**DOI:** 10.1002/anie.201511708

**Published:** 2016-01-28

**Authors:** Philippe B. Wilson, Ian H. Williams

**Affiliations:** ^1^ Department of ChemistryUniversity of BathBathBA2 7AYUK

**Keywords:** computational chemistry, enzyme catalysis, hydrogen bonds, isotope effects, methyl transfer

## Abstract

DFT calculations for methyl cation complexed within a constrained cage of water molecules permit the controlled manipulation of the “axial” donor/acceptor distance and the “equatorial” distance to hydrogen‐bond acceptors. The kinetic isotope effect *k*(CH_3_)/*k*(CT_3_) for methyl transfer within a cage with a short axial distance becomes less inverse for shorter equatorial C⋅⋅⋅O distances: a decrease of 0.5 Å results in a 3 % increase at 298 K. Kinetic isotope effects in AdoMet‐dependent methyltransferases may be m∧odulated by CH⋅⋅⋅O hydrogen bonding, and factors other than axial compression may contribute, at least partially, to recently reported isotope‐effect variations for catechol‐*O*‐methyltransferase and its mutant structures.

Quantum‐mechanical (QM) calculations for a model methyl‐transfer reaction occurring inside a constrained cage of water molecules have revealed that secondary kinetic isotope effects (2° KIEs), *k*(CH_3_)/*k*(CD_3_) and *k*(CH_3_)/*k*(CT_3_), vary significantly in response to controlled changes in CH⋅⋅⋅O interactions in the equatorial plane of the transition state (TS) for a fixed donor–acceptor distance along the methyl‐transfer axis. This finding indicates that CH⋅⋅⋅O hydrogen bonding in AdoMet‐dependent methyltransferases as noted by Trievel and co‐workers^1^ may also modulate 2° KIEs, and that factors other than axial compression might contribute, at least partially, to the intriguing KIE variations for catechol‐*O*‐methyltransferase (COMT) and its mutant structures reported recently by Klinman and co‐workers.^2^, ^3^

Besides providing the prototypical example of an S_N_2 mechanism, methyl transfer is an important component of many biological processes, not least in reactions mediated by AdoMet.^4^ In view of the small size of the methyl group, it is not obvious how an enzyme might preferentially stabilize the TS for methyl transfer relative to the reactant state (RS). The observation of an inverse D_3_ KIE of unusually large magnitude in the COMT‐catalyzed reaction of AdoMet with catecholate led to a hypothesis that catalysis might be facilitated by mechanical compression along the nucleophile/nucleofuge axis.^5^ However, hybrid QM/molecular‐mechanical (MM) computational simulations of this KIE for methyl transfer in solution and in the active site of COMT did not support the compression hypothesis: the trend in the KIEs was reproduced but without any significant difference in the average distance between the methyl donor and acceptor atoms in the corresponding TSs.^6^, ^7^ Nonetheless, an apparent trend in recent experimental T_3_ KIEs for wild‐type and mutant COMTs has been interpreted as new evidence for compression.^2^, ^3^ Meanwhile, the functional importance of unconventional CH⋅⋅⋅O hydrogen bonding in AdoMet‐dependent methyltransferases has been noted from a survey of high‐resolution crystal structures,^2^ but the possible influence of these interactions on KIEs for methyl transfer is unknown.

Herein we present results for computational investigations of the isotopic sensitivity of the methyl cation trapped inside a constrained cage (Figure 1) which permits the controlled manipulation of both the “axial” distance between donor/acceptor atoms and the “equatorial” distance to hydrogen‐bond acceptors. This model system offers access to structures not amenable to experiment but which help to provide a framework for the interpretation of KIEs that might be observed for reactions in highly structured environments, such as enzyme active sites or, potentially, within the cavities of nanoporous materials.

**Figure 1:**
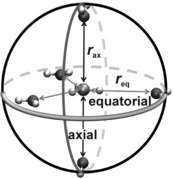
Geometry of the constrained cage complexed with methyl cation.

The cage comprises five water molecules arranged at the vertices of a trigonal bipyramid. Quasi‐*D*_3*h*_‐symmetric structures are obtained by placing a methyl cation at its center, coplanar with the three equatorial water molecules and with collinear CH⋅⋅⋅O_eq_ interactions, and perpendicular to the plane of the two axial water molecules. Each water molecule is frozen at particular fixed values of the C⋅⋅⋅O_ax_ (*r*_ax_) and C⋅⋅⋅O_eq_ (*r*_eq_) distances in the symmetric structures, and the rigid cage structure is maintained even when the methyl position is allowed to relax axially to form a tetrahedral CH_3_OH_2_^+^ RS adduct with one of the axial water molecules. All calculations were performed with the B3LYP/aug‐cc‐PVDZ density‐functional method. Isotope effects were determined within the standard rigid‐rotor harmonic‐oscillator approximation^8^ as quotients *f*_RS_/*f*_TS_, where *f* is an isotopic partition function ratio *Q*_heavy_/*Q*_light_. The KIE factor due to the three carbon–hydrogen stretching frequencies was determined by means of the Bigeleisen equation (see the Supporting Information for full details).

The D_3_ isotope effect for the transfer of methyl cation from vacuum to water, evaluated as an average over 40 solvent configurations (each a locally relaxed snapshot from a hybrid AM1/TIP3P molecular‐dynamics simulation at 298 K), is 0.85;^9^ the closest water molecules in both the axial and equatorial directions in these QM/MM structures are located at C⋅⋅⋅O distances ranging from 2.95 to 3.20 Å (i.e. close to the sum of the van der Waals radii). The transfer of methyl cation (alone) from vacuum to the center of the water cage (in which the complex is a TS with respect to methyl transfer along the axial direction with an imaginary frequency for antisymmetric C⋅⋅⋅O_ax_ stretching) similarly yields a D_3_ isotope effect of 0.86 for *r*_ax_=*r*_eq_=3.0 Å at 298 K, thus showing the reasonableness of the calculation procedures used with the cage model. However, the magnitude of this IE increases (in an inverse sense) to 0.30 for *r*_ax_=2 Å, *r*_eq_=3.0 Å because loss of methyl‐group translational and rotational motions is inadequately compensated by vibrational gains within the smaller cage.

Symmetric axial structures [H_2_O⋅⋅⋅CH_3_^+^⋅⋅⋅OH_2_] without the three equatorial water molecules (*r*_eq_=∞) possess an imaginary frequency for methyl transfer. Figure 2 shows D_3_ equilibrium isotope effects (EIEs) for the transfer of these structures from the vacuum into the center of the three‐water equatorial ring of the cage as a function of *r*_eq_ for different *r*_ax_ distances. A decrease in *r*_eq_ from 4 to 3 Å for *r*_ax_=3 Å has very little effect on the EIE (ca. 1), but the same change in *r*_eq_ for *r*_ax_=2 Å (corresponding very closely to the optimized C⋅⋅⋅O distance in the gas‐phase S_N_2 TS) inversely increases the EIE from 0.99 to 0.84. Equatorial CH⋅⋅⋅O interactions affect the EIE significantly.

**Figure 2:**
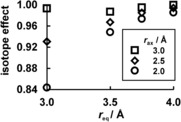
2° D_3_ EIES (298 K) for the transfer of axial [H_2_O⋅⋅⋅CH_3_^+^⋅⋅⋅OH_2_] structures from the vacuum into the center of the equatorial ring of the constrained cage.

For each *r*_eq_ distance, variation in the axial nucleophile–nucleofuge distance changes the D_3_ KIE from RS to TS within the cage dramatically from about 1.1 at *r*_ax_=2 Å to about 3 at *r*_ax_=4 Å. This increase in the D_3_ KIE corresponds to a change from a relatively tight S_N_2 TS to a very loose “exploded” S_N_2 TS.^10^, ^11^ On a per deuterium basis, these KIEs are equivalent to about 1.03 for *r*_ax_=2 Å and about 1.4 for *r*_ax_=4 Å, which are plausible values for 2° α‐D KIEs.^8^ The S_N_1‐like behavior is elicited by the imposed constraints within the cage environment; of course, such behavior is abnormal for methyl transfer, and not amenable to experimental study, but it was also seen in earlier computational studies.^12^ The KIEs calculated for methyl transfer within the water cage are all chemically reasonable, but the primary purpose of this study was to model behavior not in water but in a protein environment with hydrogen‐bond‐acceptor groups in close proximity to the methyl group. Thus, to investigate the possible influence of equatorial CH⋅⋅⋅O interactions on D_3_ KIEs within an enzyme active site, we focus upon results for *r*_ax_=2 Å within a “superheavy” constrained cage (in which each water H atom has a mass of 999 Da) to better mimic a protein environment (e.g. COMT≈30 kDa) and to remove unrealistic vibrational couplings between the methyl group and light cage H atoms.

The 2° D_3_ and T_3_ KIEs depend very significantly on the equatorial CH⋅⋅⋅O distance (Figure 3): a 0.5 Å decrease in *r*_eq_ raises the value by 2 and 3 %, respectively. The three CH bond‐stretching vibrational modes together contribute inversely to these KIEs, because the force constant *F*_CH_ increases from RS to TS.^13^ However, the respective factors (D_3_ and T_3_ CH str) diminish in magnitude (i.e. become less inverse) as *r*_eq_ decreases, because Δ*F*_CH_^≠^ also decreases as the CH⋅⋅⋅O interactions strengthen (Table 1). The CH bond‐stretching factor (which itself is dominated by changes in zero‐point energy) is responsible for the trend in the KIEs with changing *r*_eq_, whereas the overall normal direction of these isotope effects is due to all the other modes (especially bending and vibration of the methyl group within the constrained cage). The CH bonds are shorter and stiffer in TS than in RS.

**Figure 3:**
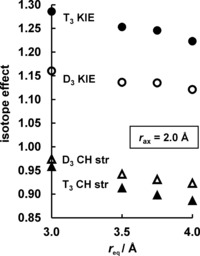
Influence of equatorial CH⋅⋅⋅O interactions (*r*_eq_) on 2° T_3_ and D_3_ KIEs (298 K) for methyl transfer within a superheavy constrained cage with *r*_ax_=2 Å, along with contributions of CH stretching modes of the complexes.

**Table 1:** Differences in the B3LYP/aug‐cc‐pVDZ barrier height, CH bond length, and CH stretching force constant between RS and TS for methyl transfer within the constrained cage, together with CH stretching factors contributing to 2° D_3_ and T_3_ KIEs at 298 K.

*r*_eq_ [Å]	3.0	3.5	3.75	4.0	∞
Δ*E* ^≠^ [kJ mol^−1^]	17.5	20.2	21.8	23.2	28.3
Δ*r*_CH_^≠^ [Å]	−0.0011	−0.0014	−0.0026	−0.0029	−0.004
Δ*F*_CH_^≠^ [aJ Å^−2^]	0.100	0.132	0.146	0.158	0.196
D_3_ CH str	0.974	0.942	0.931	0.923	
T_3_ CH str	0.958	0.913	0.898	0.886	

The strengthened CH⋅⋅⋅O hydrogen‐bonding interactions also cause the energy‐barrier height for methyl transfer within the cage to decrease from 23.2 kJ mol^−1^ at *r*_eq_=4 Å to 17.5 kJ mol^−1^ at *r*_eq_=3 Å (Table 1), equivalent to an order‐of‐magnitude increase in catalysis. Wolfe et al. reported^14^ calculations of D_3_ KIEs for intramolecular methyl transfer between O atoms confined within a rigid template, but the effect that they called transverse compression appears to have been purely repulsive in nature: there were no stabilizing interactions with hydrogen‐bond acceptors, and the transverse force distorted the TSs away from collinearity along the methyl‐transfer axis. Moliner and Williams considered D_3_ KIEs for intra‐bridgehead methyl transfer inside a symmetrical cryptand containing CH⋅⋅⋅O interactions, but this system could not be manipulated independently of the donor–acceptor distance.^15^

Although these results have been obtained for a model system, nonetheless they clearly suggest a possible role for CH⋅⋅⋅O interactions in modulating the magnitude of D_3_ and T_3_ KIEs in methyl‐transfer reactions. Use of an anionic hydrogen‐bond acceptor (e.g. carboxylate) instead of neutral water might be expected to enhance these effects. The functional importance of CH⋅⋅⋅O hydrogen bonding in AdoMet‐dependent methyltransferases has been argued,^7^ but a link to KIEs has not been previously proposed. It is known, however, that CD bond stretching frequencies are sensitive to the local electric field within a protein environment.^16^ Klinman and co‐workers have reported T_3_ KIEs on *k*_cat_/*K*_m_ in human COMT: 0.791±0.012 for the wild‐type enzyme and 0.822±0.021 and 0.850±0.012 for its Y68F and Y68A mutants, respectively.^2^, ^3^ The trend in these KIEs has been interpreted in terms of mediation of the distance between the methyl donor and acceptor groups,^3^ or “active‐site compaction”. In terms of the cage model presented herein, the variations in KIE for COMT mutants would be attributed to changes in *r*_ax_, whereas we now suggest a significant role for changes in *r*_eq_ which may lead to variations in KIE of similar magnitudes to those reported by Klinman and co‐workers. It is possible that factors other than compression along the methyl donor–acceptor axis may contribute, at least partially, to the intriguing KIE variations for COMT and its mutant structures. It has already been shown that 2° KIEs are very sensitive to the local dielectric constant.^9^ Certainly, the present results for a model methyl transfer strongly indicate the necessity for explicit inclusion of CH⋅⋅⋅O interactions in the QM region and Hessian in any new QM/MM simulations of these KIEs for COMT‐catalyzed methyl transfer.^17^ Earlier calculations^3^, ^4^ included these interactions only across the QM/MM boundary and did not include in the Hessian the Met40 and Asp141 residues, which make close contact with the methyl‐group H atoms. An electrostatic origin for catalysis in COMT^18^ and its mutants^19^ has been demonstrated and should also serve to explain trends in KIEs.

The practical utility of KIEs as part of a multidisciplinary approach to determining TS structure, and thence to designing potential drugs as TS‐analogue enzyme inhibitors, has been demonstrated.^20^ Computational modeling plays an important role within this approach, and the quality of the information it provides about TS structures for enzymatic reactions depends upon the reliability of the method used for KIE calculations in protein environments. The insight provided by the present study of the influence of CH⋅⋅⋅O interactions on 2° KIEs in methyl transfer may be of value for computational modeling not only of methyltransferases (e.g. COMT as a potential target for pancreatic‐cancer therapy)^21^ but of many other enzymes for which KIE data are available.

## Supporting information

As a service to our authors and readers, this journal provides supporting information supplied by the authors. Such materials are peer reviewed and may be re‐organized for online delivery, but are not copy‐edited or typeset. Technical support issues arising from supporting information (other than missing files) should be addressed to the authors.

Supplementary

## References

[1] S. Horowitz , L. M. A. Dirk , J. D. Yesselman , J. S. Nimtz , U. Adhikari , R. A. Mehl , S. Scheiner , R. L. Houtz , H. M. Al-Hashimi , R. C. Trievel , J. Am. Chem. Soc. 2013, 135, 15536–15548. 24093804 10.1021/ja407140k

[2] J. Zhang , J. P. Klinman , J. Am. Chem. Soc. 2011, 133, 17134–17137. 21958159 10.1021/ja207467d PMC3219439

[3] J. Zhang , H. J. Kulik , T. J. Martinez , J. P. Klinman , Proc. Natl. Acad. Sci. USA 2015, 112, 7954–7959. 26080432 10.1073/pnas.1506792112 PMC4491759

[4] A.-W. Struck , M. L. Thompson , L. S. Wong , J. Micklefield , ChemBioChem 2012, 13, 2642–2655. 23180741 10.1002/cbic.201200556

[5] M. F. Hegazi , R. T. Borchardt , R. L. Schowen , J. Am. Chem. Soc. 1979, 101, 4359–4365.

[6] G. D. Ruggiero , I. H. Williams , M. Roca , V. Moliner , I. Tuñón , J. Am. Chem. Soc. 2004, 126, 8634–8635. 15250699 10.1021/ja048055e

[7] N. Kanaan , J. J. Ruiz-Pernía , I. H. Williams , Chem. Commun. 2008, 6114–6116. 10.1039/b814212b 19082090

[8] L. Melander , W. H. Saunders , Reaction Rates of Isotopic Molecules, Wiley, New York, 1980.

[9] P. B. Wilson , P. J. Weaver , I. R. Greig , I. H. Williams , J. Phys. Chem. B 2015, 119, 802–809. 25010417 10.1021/jp505344a

[10] G. A. Craze , A. J. Kirby , R. Osborne , J. Chem. Soc. Perkin Trans. 2 1978, 357–369.

[11] B. L. Knier , W. P. Jencks , J. Am. Chem. Soc. 1980, 102, 6789–6798.

[12] G. D. Ruggiero , I. H. Williams , J. Chem. Soc. Perkin Trans. 2 2001, 448–458.

[13] I. H. Williams , J. Am. Chem. Soc. 1984, 106, 7206–7212.

[14] S. Wolfe , C.-K. Kim , K. Yang , N. Weinberg , Z. Shi , Can. J. Chem. 1998, 76, 359–370.

[15] V. Moliner , I. H. Williams , J. Am. Chem. Soc. 2000, 122, 10895–10902.

[16] M. C. Thielges , D. A. Case , F. E. Romesberg , J. Am. Chem. Soc. 2008, 130, 6597–6603. 18412341 10.1021/ja0779607 PMC2748670

[17] M. Roca, M. Sato, P. B. Wilson, V. Moliner, I. Tuñón, I. H. Williams, *manuscript in preparation*.

[18] M. Roca , S. Martí , J. Andrés , V. Moliner , I. Tuñón , J. Bertrán , I. H. Williams , J. Am. Chem. Soc. 2003, 125, 7726–7737. 12812514 10.1021/ja0299497

[19] J. Lameira , R. P. Bora , Z. T. Chu , A. Warshel , Proteins 2015, 83, 318–330. 25388538 10.1002/prot.24717 PMC4300294

[20] V. L. Schramm , ACS Chem. Biol. 2013, 8, 71–81. 23259601 10.1021/cb300631k PMC3560411

[21] W. Wu , Q. Wu , X. Hong , L. Zhou , J. Zhang , L. You , W. Wang , H. Wu , H. Sai , Y. Zhao , Cancer Sci. 2015, 106, 576–583. 25711924 10.1111/cas.12648 PMC4452158

