# Supplementary material for: Influence of Equatorial CH⋅⋅⋅O Interactions on Secondary Kinetic Isotope Effects for Methyl Transfer
Source: Angew Chem Int Ed Engl. 2016 Jan 28;55(9):3192–5. doi: 10.1002/anie.201511708 (PMC4770435; doi:10.1002/anie.201511708)
Supplement: Supplementary file 1 — Supplementary [file ANIE-55-3192-s001.pdf]

Angewandte Chemie
Eine Zeitschrift der Gesellschaft Deutscher Chemiker

Supporting Information

Influence of Equatorial CH···O Interactions on Secondary Kinetic Isotope Effects for Methyl Transfer

*Philippe B. Wilson and Ian H. Williams**

anie_201511708_sm_miscellaneous_information.pdf

Supporting Information

Contents **page**

Computational details 2

References 4

Table S1. Z-matrix template for TS cage complexes 5

Table S2. Optimised parameters for cage transition structures 6

Note on Table S2 6

Table S3. Z-matrix template for RS cage complexes 7

Table S4. Optimised parameters for cage reactant structures 8

Notes on Table S4 8

Table S5. B3LYP/aug-cc-pVDZ optimised energies for RS and TS structures (total energies in hartree, relative energies in kJ mol^{-1}). 9

Notes on Table S5 9

Table S6. Vibrational wavenumbers (cm^{-1}) for symmetric and *antisymmetric* (italics) axial C-O bond stretching in transition structures of methyl cation in constrained water cages 10

Notes on Table S6 10

Table S7. Imaginary vibrational wavenumbers (cm^{-1}) for motions of constrained cage water molecules. Vibrations in brackets correspond to the heavier isotopologue, vibrations in regular type to the TS, and in bold to the RS 11

Notes on Table S7 14

Table S8. B3LYP/aug-cc-pVDZ average CH-bond stretching force constants (aJ $Å^{-2}$) for RS and TS.

Notes on Table S8 15

Table S9. B3LYP/aug-cc-pVDZ isotopic partition function ratios, 2° α-D_3 isotope effects for transfer of methyl cation from vacuum to the center of the constrained water cage with r_{eq} = 3.0 Å at 298 K and isotope effect factors. 16

Notes on Table S9 16

Table S10. B3LYP/aug-cc-pVDZ isotopic partition function ratios, 2° α-D_3 isotope effects for insertion of symmetric axial structures [$H_2O…CH_3^+…OH_2$] from vacuum to the center of the constrained three-water equatorial cage at 298 K and isotope effect factors. 17

Notes on Table S10 17

Table S11. B3LYP/aug-cc-pVDZ isotopic partition function ratios and factors for RS and TS, and KIEs, for methyl transfer within a constrained superheavy cage with r_{ax} = 2.04 Å. 18

Notes on Table S11. 19

Computational Methods

Constrained geometry optimizations and analytical second-derivative calculations of Hessians were performed by means of the Gaussian09 program (revision A.02).[1] The B3LYP density functional was used with the aug-cc-PVDZ basis set, a method previously identified as performing well for a harmonic vibrational frequencies, leading to reliable estimates of isotope effects.[2] Residual translational and rotational contributions to Hessian elements (punch=derivatives) were removed by a projection method[3] and equilibrium and/or kinetic isotope effects at 298.15 K were determined within the rigid-rotor/harmonic approximation without scaling of vibrational frequencies, which satisfied the Teller-Redlich product rule; tunneling was treated by means of Bell's model applied to the imaginary frequency for motion along the reaction coordinate.[4]

The conventional transition-state theory treatment of KIEs with the Born-Oppenheimer, rigid-rotor and harmonic oscillator approximations (and neglecting tunnelling and transmission-factor contributions) considers the rate-constant ratio k/k' (where the prime denotes the rate constant for the heavier isotopologue) as the product of three factors (eq. 1) involving translational, rotational and vibrational partition functions for the isotopologous RS and TS species.[4,5] The first is the mass and moments-of-inertia factor (MMI), where $|\boldsymbol{I}|$ is the determinant of the moment-of-inertia tensor (*i.e.* the product of the three principal moments of inertia) of a non-linear N-atomic molecule of molecular mass M; rotational symmetry factors are omitted here since they are all equal to unity for the applications to be presented below. The second is the Boltzmann excitational (EXC) factor corresponding to the relative populations of the higher vibrational quantum states, and the third is the zero-point energy (ZPE). EXC involves a product, and ZPE a summation, over $3N - 6$ and $3N - 7$ real vibrational frequencies of RS and TS, respectively, where $u = hc\nu/k_BT$ with frequency (s^{-1}) replaced by wavenumber ν (cm^{-1}) and h, k_B, c and T are the Planck and Boltzmann constants, the velocity of light and the absolute temperature. Inspection of the forms of the MMI, EXC and ZPE factors shows that the KIE may be rewritten simply as a quotient of isotopic partition function ratios (IPFRs) for RS and TS (eq. 2).

$$k/k' = \frac{\left[\frac{|\boldsymbol{I}'|}{|\boldsymbol{I}|}\right]^{1/2}_{RS}\left[\frac{M'}{M}\right]^{3/2}_{RS}\prod_i^{3N-6}\left[\frac{[1-\exp(-u_i)]}{[1-\exp(-u_i')]}\frac{\exp(u_i/2)}{\exp(u_i'/2)}\right]_{RS}}{\left[\frac{|\boldsymbol{I}'|}{|\boldsymbol{I}|}\right]^{1/2}_{TS}\left[\frac{M'}{M}\right]^{3/2}_{TS}\prod_i^{3N-7}\left[\frac{[1-\exp(-u_i)]}{[1-\exp(-u_i')]}\frac{\exp(u_i/2)}{\exp(u_i'/2)}\right]_{TS}} \quad (1)$$

$$\text{KIE} = \text{MMI} \times \text{EXC} \times \text{ZPE}$$

$$k/k' = f_{RS} / f_{TS} \quad (2)$$

It is common to replace MMI in eq. 1 by an equivalent factor containing only vibrational frequencies. According to the Teller-Redlich product rule,[6] the masses and moments of inertia for a pair of isotopologues are related to the vibrational frequencies: the equality expressed by eq. 3 assumes separability of translational and rotational motions from vibrational motions within the harmonic approximation. Substituting the vibrational product VP for each of RS and for TS in eq. 1,

the KIE may be written as eq. 4, the Bigeleisen equation, where VPR is the vibrational product ratio.[7,8] Two points should be noted: (i) the product of ratios of atomic masses *m* that appears eq. 3 vanishes from the KIE because it is identical for both RS and TS; (ii) it is conventional to consider the ratio of imaginary transition frequencies for the TS as a separate factor, so that (in eq. 5) the VPR and EXC products and the ZPE summation are all taken over $3N - 7$ real TS frequencies.

$$\prod_{i}^{3N-6} \frac{\nu_i'}{\nu_i} \;=\; \mathrm{VP} \;=\; \mathrm{MI} \;=\; \left[\frac{|\boldsymbol{I}'|}{|\boldsymbol{I}|}\right]^{1/2} \left[\frac{M'}{M} \prod_{j}^{N} \frac{m_j}{m_j'}\right]^{3/2} \qquad (3)$$

$$k/k' \;=\; (\nu_{\ddagger} / \nu_{\ddagger}') \times \mathrm{VPR} \times \mathrm{EXC} \times \mathrm{ZPE} \qquad (4)$$

$$\mathrm{VPR} \;=\; \prod_{i}^{3N-6}\left[\frac{\nu_i'}{\nu_i}\right]_{\mathrm{RS}} \Big/ \prod_{i}^{3N-7}\left[\frac{\nu_i'}{\nu_i}\right]_{\mathrm{TS}} \qquad (5)$$

Use of the Bigeleisen equation enables the determination of KIEs from normal-mode frequencies without the need to consider the MMI factor explicitly. However, there is practical merit in separately determining the vibrational product and mass-moment-of-inertia terms for a pair of isotopologues, rather than relying upon their equality: the requirement for VP and MI to be equal in value provides a strict test for the correctness of the vibrational frequency calculations. If these two terms are not equal to a satisfactory number of decimal places, it is a sure indication that something is wrong, often (in our experience) with the definition of internal coordinates used in the projection method for removing residual components of translational and rotational motion from the Hessian.

We prefer to evaluate IPFRs for each RS or TS species separately and then to evaluate kinetic isotope effects (KIEs) by means of eq. 2 or equilibrium isotope effects (EIEs) by the analogous eq. 6, where PS refers to a minimum-energy product structure.

$$K/K' \;=\; f_{\mathrm{RS}} / f_{\mathrm{PS}} \qquad (6)$$

Within the inherent assumption of separability in this treatment of KIEs, the quantum correction $\Gamma_{\ddagger}/\Gamma_{\ddagger}'$ for motion along the transition vector may be approximated by Bell's expression[9] for an inverted parabola, eq. 7; this quantum correction is applied routinely to a transition frequency in evaluation of the IPFR for isotopologous TSs.

$$\frac{\Gamma_{\ddagger}}{\Gamma_{\ddagger}'} \;=\; \frac{\nu_{\ddagger}}{\nu_{\ddagger}'} \frac{\sin(u_i'/2)}{\sin(u_i/2)} \qquad (7)$$

References

1. M. J. Frisch, G. W. Trucks, H. B. Schlegel, G. E. Scuseria, M. A. Robb, J. R. Cheeseman, G. Scalmani, V. Barone, B. Mennucci, G. A. Petersson, H. Nakatsuji, M. Caricato, X. Li, H. P. Hratchian, A. F. Izmaylov, J. Bloino, G. Zheng, J. L. Sonnenberg, M. Hada, M. Ehara, K. Toyota, R. Fukuda, J. Hasegawa, M. Ishida, T. Nakajima, Y. Honda, O. Kitao, H. Nakai, T. Vreven, J. A. Montgomery, Jr., J. E. Peralta, F. Ogliaro, M. Bearpark, J. J. Heyd, E. Brothers, K. N. Kudin, V. N. Staroverov, R. Kobayashi, J. Normand, K. Raghavachari, A. Rendell, J. C. Burant, S. S. Iyengar, J. Tomasi, M. Cossi, N. Rega, J. M. Millam, M. Klene, J. E. Knox, J. B. Cross, V. Bakken, C. Adamo, J. Jaramillo, R. Gomperts, R. E. Stratmann, O. Yazyev, A. J. Austin, R. Cammi, C. Pomelli, J. W. Ochterski, R. L. Martin, K. Morokuma, V. G. Zakrzewski, G. A. Voth, P. Salvador, J. J. Dannenberg, S. Dapprich, A. D. Daniels, O. Farkas, J. B. Foresman, J. V. Ortiz, J. Cioslowski, and D. J. Fox, *Gaussian 09*, Revision A.02 (Bath) and Revision B.01 (Rikkyo), Gaussian, Inc.: Wallingford CT, **2009**.
2. P. B. Wilson, I. H. Williams, *Mol. Phys*. **2015**, *113*, 1704-1711.
3. (a) I. H. Williams, *Chem. Phys. Lett.* **1982**, *88*, 462-466. (b) I. H. Williams, *J. Mol. Struct. THEOCHEM* **1983**, *11*, 275-284.
4. E. K. Thornton, E. R. Thornton, in *Isotope Effects in Chemical Reactions*, (Eds.: C. J. Collins, N. S. Bowman), Van Nostrand Reinhold, New York, **1970**.
5. L. Melander, W. H. Saunders, *Reaction Rates of Isotopic Molecules*, Wiley, New York, **1980**, pp. 71–73.
6. (a) E. Teller, *cf*. W. R. Angus, C. R. Bailey, J. B. Hale, C. K. Ingold, A. H. Leckie, C. G. Raisin, J. W. Thompson, C. L. Wilson, *J. Chem. Soc.* **1936**, 971-987. (b) O. Redlich, *Z. Physik. Chem. B* **1935**, *28*, 371-382.
7. (a) M. Wolfsberg, M. Stern, J. *Pure Appl. Chem*. **1964**, *8*, 225-242. (b) M. J. Stern, M. Wolfsberg, *J. Chem. Phys*. **1966**, *45*, 4105-4124.
8. (a) J. Bigeleisen, M. G. Mayer, *J. Chem. Phys*. **1947**, *15*, 261-267. (b) J. Bigeleisen, *J. Chem. Phys*. **1949**, *17*, 675-678.

9 R. P. Bell, *Trans. Faraday Soc*. **1959**, *55*, 1-4.

10 P. B. Wilson, P. J. Weaver, I. R. Greig, I. H. Williams, *J. Phys. Chem. B*. **2015**, *119*, 802-809.

11 G. D. Ruggiero, I. H. Williams, *J. Chem. Soc., Perkin Trans. 2* **2002**, 591-597.

Table S1. Z-matrix template for TS cage complexes.

C						
X	1	c_1				
X	2	c_1	1	c_90		
H	1	v_CH	2	c_90	3	c_0
H	1	v_CH	2	c_90	3	c_120
H	1	v_CH	2	c_90	3	- c_120
O	1	c_Rax	4	c_90	3	c_0
X	7	c_1	1	c_90	4	c_0
X	7	c_1	8	c_90	1	c_180
H	7	v_HOax	9	v_Aax	8	c_90
H	7	v_HOax	9	v_Aax	8	- c_90
O	1	c_Rax	4	c_90	3	c_180
X	12	c_1	1	c_90	4	c_0
X	12	c_1	13	c_90	1	c_180
H	12	v_HOax	14	v_Aax	13	c_90
H	12	v_HOax	14	v_Aax	13	- c_90
O	1	c_Req	2	c_90	3	c_0
X	17	c_1	1	c_90	2	c_0
X	17	c_1	18	c_90	1	c_180
H	17	v_HOeq	19	v_Aeq	18	c_90
H	17	v_HOeq	19	v_Aeq	18	- c_90
O	1	c_Req	2	c_90	3	c_120
X	22	c_1	1	c_90	2	c_0
X	22	c_1	23	c_90	1	c_180
H	22	v_HOeq	24	v_Aeq	23	c_90
H	22	v_HOeq	24	v_Aeq	23	- c_90
O	1	c_Req	2	c_90	3	- c_120
X	27	c_1	1	c_90	2	c_0
X	27	c_1	28	c_90	1	c_180
H	27	v_HOeq	29	v_Aeq	28	c_90
H	27	v_HOeq	29	v_Aeq	28	- c_90

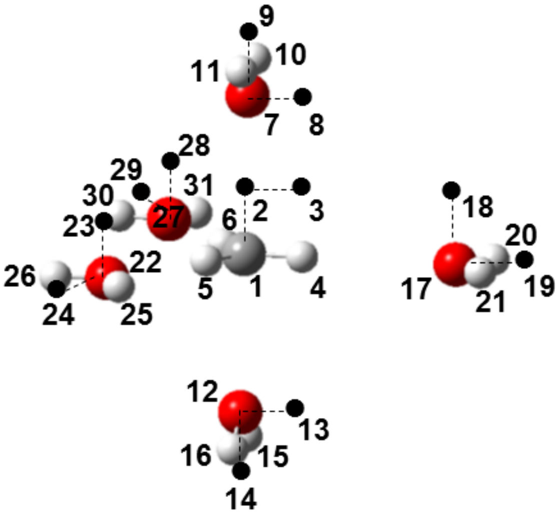

Variables:

v_CH 1.1014
v_HOax0.9664
v_Aax 55.0
v_HOeq0.9656
v_Aeq 54.74

Constants:

c_Rax 2.04 ! this is r_{ax} in the paper
c_Req 3.0 ! this is r_{eq} in the paper
c_0 0.0
c_1 1.0
c_90 90.0
c_120 120.0
c_180 180.0

Table S2. Optimised parameters for cage transition structures.

Rax / Å	Req / Å	CH / Å	HOax / Å	Aax / °	HOeq / Å	Aeq / °	HCOax / °
2.04	3.0	1.08499	0.96469	54.6395	0.96596	52.277	90.0
	3.5	1.08817	0.96528	54.7945	0.96552	52.300	90.0
	3.75	1.08750	0.96554	54.806	0.96551	52.2795	90.0
	4.0	1.08667	0.96576	54.800	0.96553	52.251	90.0
2.525	3.0	1.09642	0.96467	53.7425	0.96575	52.4315	90.0
	3.5	1.09693	0.96498	53.9960	0.96542	52.3712	90.0
	3.75	1.09561	0.96515	54.0535	0.96544	52.3275	90.0
	4.0	1.09436	0.96530	54.0862	0.96547	52.2835	90.0
3.0	3.0	1.10304	0.96505	52.9624	0.96574	52.5279	90.0
	3.5	1.10206	0.96519	53.1986	0.96541	52.4223	90.0
	3.75	1.10038	0.96528	53.2690	0.96543	52.364	90.0
	4.0	1.09890	0.96536	53.3171	0.96546	52.3084	90.0

Note on Table S2:

The main text refers to values of r_{ax} = 2.0, 2.5 and 3.0 Å. In fact the precise values used in this study were r_{ax} = 2.04, 2.525 and 3.0 Å. The former two values are a legacy from an earlier published study on methyl cation within a continuum solvation model:[10] 2.04 Å is the PCM default radius for a carbon atom within the UFF cavity model, and 2.525 Å is the default radius for a CH_3 group within the UA0 cavity model.

Table S3. Z-matrix template for RS cage complexes.

O						
C	1	v_CO				
H	2	v_CH	1	v_OCH		
H	2	v_CH	1	v_OCH	3	c_120
H	2	v_CH	1	v_OCH	3	- c_120
H	1	v_HOax	2	c_Aax	3	c_90
H	1	v_HOax	2	c_Aax	3	- c_90
O	1	c_OO	6	c_Aax	2	c_0
H	8	v_HOax	2	c_Aax	3	c_90
H	8	v_HOax	2	c_Aax	3	- c_90
X	1	c_Rax	6	c_Aax	2	c_0
O	11	c_Req	1	c_90	3	c_0
H	12	c_HOeq	11	c_Aeq	1	c_90
H	12	c_HOeq	11	c_Aeq	1	- c_90
O	11	c_Req	1	c_90	3	c_120
H	15	c_HOeq	11	c_Aeq	1	c_90
H	15	c_HOeq	11	c_Aeq	1	- c_90
O	11	c_Req	1	c_90	3	- c_120
H	18	c_HOeq	11	c_Aeq	1	c_90
H	18	c_HOeq	11	c_Aeq	1	- c_90

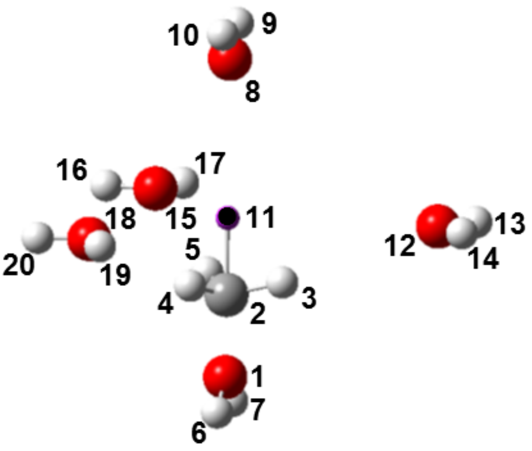

Variables:

v_CO 1.56677

v_CH 1.08504

v_HCax 102.0

Constants:

c_OO 4.08 ! this is r_{ax} x 2

c_Rax 2.04 ! this is r_{ax} in the paper

c_Req 3.0 ! this is r_{eq} in the paper

c_HOax0.96469

c_Aax125.361

c_HOeq0.96596

c_Aeq127.723

c_0 0.0

c_90 90.0

c_120 120.0

Table S4. Optimised parameters for cage reactant structures.

Rax / Å	Req / Å	CH / Å	HOax / Å	COHax / °	HOeq / Å	COHeq / °	HCOax / °
2.04	3.0	1.08609	0.96469	125.361	0.96596	127.723	102.249
	3.5	1.09039	0.96469	125.361	0.96596	127.723	102.825
	3.75	1.09010	0.96469	125.361	0.96596	127.723	103.174
	4.0	1.08959	0.96469	125.361	0.96596	127.723	103.428
2.525	3.0	1.09287	0.96469	125.361	0.96596	127.723	103.831
	3.5	1.09420	0.96469	125.361	0.96596	127.723	104.185
	3.75	1.09359	0.96469	125.361	0.96596	127.723	104.375
	4.0	1.09289	0.96469	125.361	0.96596	127.723	104.552
3.0	3.0	1.09513	0.96469	125.361	0.96596	127.723	104.472
	3.5	1.09452	0.96469	125.361	0.96596	127.723	104.631
	3.75	1.09384	0.96469	125.361	0.96596	127.723	104.713
	4.0	1.09322	0.96469	125.361	0.96596	127.723	104.811

Note on Table S4:

The main text refers to values of r_{ax} = 2.0, 2.5 and 3.0 Å. In fact the precise values used in this study were r_{ax} = 2.04, 2.525 and 3.0 Å. The former two values are a legacy from an earlier published study on methyl cation within a continuum solvation model:[10] 2.04 Å is the PCM default radius for a carbon atom within the UFF cavity model, and 2.525 Å is the default radius for a CH_3 group within the UA0 cavity model.

Table S5. B3LYP/aug-cc-pVDZ optimised energies for RS and TS structures (total energies in hartree, relative energies in kJ mol^{-1}).

		r_{eq} / Å				
r_{ax} / Å		3.0	3.5	3.75	4.0	∞
2.04	E_{RS}	-421.86098	-421.86284	-421.85963	-421.85637	-192.49373
	E_{TS}	-421.85431	-421.85515	-421.85132	-421.84755	-192.48296
	$\Delta E^{\ddagger}$	17.5	20.2	21.8	23.2	28.3
2.525	E_{RS}	-421.85795	-421.85585	-421.85265	-421.84965	-192.48948
	E_{TS}	-421.83256	-421.82884	-421.82388	-421.81939	-192.45370
	$\Delta E^{\ddagger}$	66.6	70.9	75.5	79.4	93.9
3.0	E_{RS}	-421.85239	-421.84793	-421.84511	-421.84261	-192.48528
	E_{TS}	-421.81262	-421.80504	-421.79898	-421.79371	-192.42612
	$\Delta E^{\ddagger}$	104	113	121	128	155

Notes on Table S5:

$\Delta E^{\ddagger}$ is the barrier height for methyl transfer between axial waters within the constrained cage, either with the three equatorial waters present (r_{eq} = 3.0, 3.5, 3.75 and 4.0 Å) or without them ($r_{eq} = \infty$). On the one hand, the value of this barrier increases steeply as the methyl-transfer distance (= 2 × r_{ax}) increases; this may be rationalized simply in terms of the Principle of Least Nuclear Motion, as has been noted previously.[11] On the other hand, the value of this barrier decreases as the CH…O distance (r_{eq}) decreases. This reflects a differential between the RS and TS energies for interaction between the each of the symmetric axial structures [$H_2O…CH_3^+…OH_2$] and the three equatorial water molecules which serves to stabilize the TS more effectively for shorter, stronger CH…O hydrogen-bonding interactions. (These interactions give rise to catalysis within the constrained cage model.)

Table S6. Vibrational wavenumbers (cm^{-1}) for symmetric and *antisymmetric* (italics) axial C-O bond stretching in transition structures of methyl cation in constrained water cages.

r_{ax} / Å		r_{eq} / Å									
		3.0		3.5		3.75		4.0		∞	
		CH_3	CD_3	CH_3	CD_3	CH_3	CD_3	CH_3	CD_3	CH_3	CD_3
2.04	S	258	258	260	260	259	259	260	260	263	262
	A	339*i*	337*i*	357*i*	355*i*	363*i*	361*i*	367*i*	366*i*	385*i*	380*i*
2.525	S	82*i*	*82i*	85*i*	*85i*	84*i*	*84i*	83*i*	*83i*	78*i*	78*i*
	A	258*i*	248*i*	268*i*	259*i*	273*i*	264*i*	276*i*	267*i*	285*i*	275*i*
3.0	S	92*i*	92*i*	98*i*	98*i*	99*i*	99*i*	99*i*	99*i*	97*i*	97*i*
	A	190*i*	181*i*	197*i*	188*i*	202*i*	192*i*	206*i*	196*i*	216*i*	205*i*
**Superheavy cage** (all H atoms of "water" molecules have mass = 999):											
2.04	S	22	22	22	22	22	22	22	22		
	A	264*i*	257*i*	279*i*	273*i*	286*i*	280*i*	291*i*	285*i*		

Notes on Table S6:

The antisymmetric stretching mode is the reaction-coordinate mode (or transition vector) for methyl transfer within the cage. Its frequency is imaginary (as expected) in all cases. Replacement of protium in CH_3 by deuterium in CD_3 leads to a diminution in magnitude of this frequency (as expected). An increase either in the value of r_{eq} for a particular value of r_{ax} or in the value of r_{ax} for a particular value of r_{eq} leads to an increase in the magnitude of this frequency, consistent with an increase in the barrier height $\Delta E^{\ddagger}$ for methyl transfer (see Table S5).

The symmetric stretching frequency is insensitive to isotopic substitution in the methyl group because atomic motions of these atoms do not contribute to this normal mode of vibration. This frequency has a real value for r_{ax} = 2.04 Å but is imaginary for r_{ax} = 2.525 and 3.0 Å. However, it is important to recognise that these symmetric structures do not correspond to stationary points on the potential energy surface for the full unconstrained system: in particular, the gradient of the energy with respect to displacement in the symmetric stretching coordinate is not zero.

In the "superheavy" cage, all frequencies are lowered and the isotopically-sensitive modes of the methyl group are decoupled from those of the cage environment.

Table S7. Imaginary vibrational wavenumbers (cm^{-1}) for motions of constrained cage water molecules. (Regular font correspond to the TS and **bold to the RS**.)

r_{ax} / Å		r_{eq} / Å									
		3.0		3.5		3.75		4.0		∞	
	description	CH_3	CD_3	CH_3	CD_3	CH_3	CD_3	CH_3	CD_3	CH_3	CD_3
2.04	ax-wag	418*i*	417*i*	419*i*	418*i*	416*i*	415*i*	412*i*	411*i*	369*i*	368*i*
		**496*i***	**496*i***	**496*i***	**495*i***	**495*i***	**495*i***	**495*i***	**494*i***	**479*i***	**478*i***
	ax-wag	403*i*	403*i*	408*i*	408*i*	406*i*	405*i*	403*i*	402*i*	363*i*	362*i*
		**295*i***	**295*i***	**280*i***	**279*i***	**274*i***	**274*i***	**269*i***	**269*i***	**137*i***	**137*i***
	ax-twist	146*i*	146*i*	122*i*	122*i*	113*i*	113*i*	105*i*	105*i*	82*i*	82*i*
		**393*i***	**393*i***	**422*i***	**422*i***	**431*i***	**431*i***	**438*i***	**438*i***	**384*i***	**364*i***
	ax-twist	107*i*	107*i*	92*i*	92*i*	88*i*	88*i*	83*i*	83*i*	69*i*	62*i*
		**54*i***	**53*i***								
	ax-transln.	56*i*	55*i*	34*i*	34*i*	48*i*	47*i*				
		**61*i***	**60*i***	**16*i***	**16*i***	**35*i***	**35*i***	**48*i***	**47*i***		
	eq-wag	38*i*	38*i*			27*i*	27*i*	42*i*	41*i*		
		**39*i***	**39*i***			**26*i***	**26*i***	**40*i***	**39*i***		
	eq-scissor	36*i*	34*i*			18*i*	18*i*	35*i*	35*i*		
		**34*i***	**34*i***			**20*i***	**20*i***	**34*i***	**34*i***		
	flexing	32*i*	32*i*								
		**22*i***	**22*i***	**44*i***	**44*i***	**46*i***	**45*i***	**50*i***	**50*i***		
	rotation										
								**4*i***	**4*i***		
	MI	21.44705286		21.48090880		21.49458282		21.50631983		16.25090291	
		**21.45034410**		**21.48382664**		**21.49737106**		**21.50892768**		**16.32865153**	
	VP	21.44705287		21.48090880		21.49458279		21.50631972		16.25090292	
		**21.45034411**		**21.48382704**		**21.49737148**		**21.50892671**		**16.32865153**	

Continued on next page…

Table S7. *continued*

r_{ax} / Å		r_{eq} / Å									
		3.0		3.5		3.75		4.0		∞	
	description	CH_3	CD_3	CH_3	CD_3	CH_3	CD_3	CH_3	CD_3	CH_3	CD_3
2.525	ax-wag	348*i*	347*i*	384*i*	383*i*	394*i*	392*i*	399*i*	398*i*	390*i*	389*i*
		**500*i***	**499*i***	**502*i***	**501*i***	**502*i***	**501*i***	**502*i***	**501*i***	**482*i***	**481*i***
	ax-wag	342*i*	341*i*	380*i*	380*i*	389*i*	388*i*	394*i*	393*i*	376*i*	374*i*
		**303*i***	**303*i***	**298*i***	**298*i***	**296*i***	**296*i***	**294*i***	**294*i***	**191*i***	**191*i***
	ax-twist	123*i*	123*i*	101*i*	101*i*	93*i*	93*i*	85*i*	86*i*	60*i*	60*i*
		**48*i***	**47*i***								
	ax-twist	85*i*	85*i*	72*i*	72*i*	70*i*	69*i*	68*i*	67*i*	43*i*	38*i*
		**416*i***	**416*i***	**442*i***	**442*i***	**451*i***	**451*i***	**457*i***	**457*i***	**402*i***	**381*i***
	eq-twist										
		**73*i***	**73*i***			**34*i***	**34*i***	**60*i***	**56*i***	**56*i***	**50*i***
	sym-stretch										
		**57*i***	**57*i***			**54*i***	**53*i***	**54*i***	**53*i***	**51*i***	**50*i***
	eq-stretch					52*i*	52*i*	59*i*	58*i*		
						**48*i***	**47*i***	**51*i***	**50*i***		
	eq-stretch					49*i*	48*i*	54*i*	53*i*		
						**46*i***	**45*i***	**45*i***	**44*i***		
	eq-stretch					29*i*	29*i*	40*i*	40*i*		
		**22*i***	**22*i***	**27*i***	**27*i***	**28*i***	**30*i***	**39*i***	**39*i***		
	MI	21.45869210		21.48940694		21.50172858		21.51231609		16.27344552	
		**21.43062013**		**21.46510757**		**21.47885354**		**21.49083006**		**16.35852703**	
	VP	21.45869201		21.48940646		21.50172858		21.51231604		16.27344552	
		**21.43062013**		**21.46511162**		**21.47898918**		**21.49082882**		**16.35852703**	

Continued on next page…

Table S7. *continued*

r_{ax} / Å		r_{eq} / Å									
		3.0		3.5		3.75		4.0		∞	
	description	CH_3	CD_3	CH_3	CD_3	CH_3	CD_3	CH_3	CD_3	CH_3	CD_3
3.0	wag	234*i*	233*i*	311*i*	310*i*	339*i*	336*i*	357*i*	356*i*	416*i*	415*i*
		**500*i***	**499*i***	**501*i***	**501*i***	**502*i***	**501*i***	**502*i***	**501*i***	**480*i***	**497*i***
	wag	227*i*	227*i*	309*i*	309*i*	335*i*	335*i*	354*i*	353*i*	411*i*	408*i*
		**308*i***	**308*i***	**307*i***	**307*i***	**306*i***	**306*i***	**305*i***	**305*i***	**218*i***	**218*i***
	twist	105*i*	105*i*	89*i*	89*i*	82*i*	82*i*	76*i*	76*i*	51*i*	51*i*
		**65*i***	**65*i***								
	twist	64*i*	63*i*	58*i*	58*i*	55*i*	55*i*	52*i*	52*i*	34*i*	31*i*
		**430*i***	**429*i***	**451*i***	**451*i***	**458*i***	**458*i***	**464*i***	**464*i***	**408*i***	**386*i***
	sym-stretch										
		**34*i***	**34*i***	**35*i***	**35*i***	**36*i***	**36*i***	**36*i***	**36*i***	**36*i***	**36*i***
	eq-stretch			32*i*	32*i*	67*i*	66*i*	70*i*	68*i*		
				**43*i***	**43*i***	**42*i***	**39*i***	**48*i***	**47*i***		
	eq-stretch			29*i*	28*i*	66*i*	65*i*	69*i*	67*i*		
				**46*i***	**46*i***	**52*i***	**51*i***	**47*i***	**47*i***		
	eq-stretch			41*i*	41*i*	46*i*	46*i*				
				**43*i***	**43*i***	**40*i***	**40*i***				
	flexing	29*i*	29*i*	16*i*	16*i*	16*i*	16*i*	17*i*	17*i*		
				**19*i***	**19*i***						
	MI	21.46950873		21.49761555		21.50879727		21.51839000		16.27623938	
		**21.38917652**		**21.42520186**		**21.43988590**		**21.45327838**		**16.34825736**	
	VP	21.46950873		21.49761553		21.50879728		21.51839001		16.27623935	
		**21.38917704**		**21.42520188**		**21.43988594**		**21.45327900**		**16.34825736**	

Notes on Table S7.

Owing to the fact (mentioned above in the notes on Table S6) that neither the RS nor the TS structures correspond to stationary points on the potential energy surface for the full unconstrained system, and also because of the imposed symmetry, there are a number of additional modes associated with imaginary vibrational frequencies. Four of these are evident in the $r_{eq} = \infty$ structures without the three equatorial waters, and involve in-phase and out-of-phase combinations of wagging and twisting of the axial water molecules. It is obvious that the $CH_3OH_2^+$ fragment of the constrained RS structures is unstable with respect to displacement towards a pyramidalized sp^3 O atom instead of the trigonal-planar geometry imposed by the constraints, an this instability is manifest in both the RS and TS cage structures. The constrained structures represent saddle points for inversion and torsion of the axial OH_2 moieties. However, in practice these modes are almost insensitive to isotopic substitution in the methyl group and therefore do not affect the calculated KIEs.

In the cage structures with r_{eq} = 3.0, 3.5, 3.75 and 4.0 Å, some of the modes involving the axial waters are strongly coupled with motions of the equatorial waters and the brief descriptions given in the Table are rather approximate. Imaginary frequencies are associated with some displacements the equatorial waters away from their constrained positions. When r_{ax} = 2.04 Å, the [$H_2O\ldots CH_3^+\ldots OH_2$] fragment has no room for lateral motion with respect to the equatorial waters, but for r_{ax} = 2.525 and 3.0 Å, combinations of CH…O hydrogen-bond stretching motions are associated with imaginary frequencies: these modes may also be considered as translations of [$H_2O\ldots CH_3^+\ldots OH_2$] within the equatorial plane. The important observation, however, is that in practice these modes are almost insensitive to isotopic substitution in the methyl group and therefore do not affect the calculated KIEs.

The quantities MI and VP represent the "masses & moments of inertia" and "vibrational product" terms appearing in eq.3. According to the Teller-Redlich Product Rule,[6] these terms should be equal in value. The extent to which this is found to be true in practice provides a stern test of the correctness and internal consistency of the vibrational frequency calculations, and especially of the method employed to project out contaminating components of translation and rotation from the vibrational degrees of freedom. Inspection of the results presented in Table S7 shows agreement in all cases to the fourth decimal place, with the majority being to the seventh. *This excellent agreement is achieved only if the vibrational products are evaluated by treating all the imaginary frequencies as if they were real*. It also serves to confirm our view that *the presence of imaginary frequencies associated with vibrational modes of the constrained water molecules does not have any material influence upon the values (or indeed the validity) of the calculated KIEs.*

Table S8. B3LYP/aug-cc-pVDZ average CH-bond stretching force constants (aJ Å^{-2}) for RS and TS.

		r_{eq} / Å				
r_{ax} / Å		3.0	3.5	3.75	4.0	∞
2.04	$(CH)_{RS}$	5.763	5.620	5.633	5.654	5.730
	$(CH)_{TS}$	5.863	5.752	5.779	5.812	5.926
	$\Delta^{\ddagger}$	0.100	0.132	0.146	0.158	0.196
	$\Delta\Delta^{\ddagger}_{eq}$	-0.096	-0.064	-0.050	-0.038	
2.525	$(CH)_{RS}$	5.539	5.502	5.524	5.549	5.626
	$(CH)_{TS}$	5.543	5.540	5.590	5.636	5.779
	$\Delta^{\ddagger}$	0.004	0.038	0.066	0.087	0.153
	$\Delta\Delta^{\ddagger}_{eq}$	-0.149	-0.115	-0.087	-0.066	
3.0	$(CH)_{RS}$	5.467	5.496	5.518	5.540	5.600
	$(CH)_{TS}$	5.320	5.376	5.440	5.495	5.654
	$\Delta^{\ddagger}$	-0.147	-0.120	-0.078	-0.045	0.054
	$\Delta\Delta^{\ddagger}_{eq}$	-0.201	-0.174	-0.132	-0.099	

Notes on Table S8.

The environment of three methyl CH bonds within the constrained cage does not have strict 3-fold symmetry along the axial direction and so the individual CH stretching force constants are not quite equal. The values presented in the Table are averages of the individual values. For a particular value of r_{ax}, the value of the CH stretching force constant F tends to diminish with decreasing r_{eq}, except for a slight upturn at r_{eq} = 3.0 Å for the shorter r_{ax} distances. As r_{ax} increases, the value of F decreases for both RS and TS, but more rapidly for TS, for all values of r_{eq}. Consequently the difference $\Delta^{\ddagger}$ between RS and TS changes from a positive number for r_{ax} = 2.04 Å (stiffer bond in TS) to a negative number for r_{ax} = 2.525 and 3.0 Å (looser bond in TS). The double difference $\Delta\Delta^{\ddagger}$ (between $\Delta^{\ddagger}$ values for each r_{eq} distance and $r_{eq} = \infty$) becomes more negative, monotonically, as r_{eq} decreases.

Table S9. B3LYP/aug-cc-pVDZ isotopic partition function ratios, 2° α-D_3 isotope effects for transfer of methyl cation from vacuum to the center of the constrained water cage with r_{eq} = 3.0 Å at 298 K and isotope effect factors.

$f_{vacuum}(Me^+)$	20823				
r_{ax} / Å	$f_{complex}$	KIE	MMI	EXC	ZPE
2.04	69189	0.301	3.536	0.727	0.117
2.525	32891	0.633	3.538	0.669	0.269
3.0	24213	0.860	3.540	0.607	0.401

Notes on Table S9

All hydrogen atoms have mass m_H = 1. Because each complex with the methyl cation in the center of the cage is a TS, these isotope effects are KIEs which include the quantum correction to the isotopic sensitivity of the transition frequency for methyl transfer. The normal MMI factor is almost constant. The inverse EXC and ZPE factors show opposite trends, but the latter dominates and is primarily responsible for the trend for a larger (more inverse) isotope effect with decreasing r_{ax} distance.

Table S10. B3LYP/aug-cc-pVDZ isotopic partition function ratios, 2° α-D_3 isotope effects for insertion of symmetric axial structures [$H_2O…CH_3^+…OH_2$] from vacuum to the center of the constrained three-water equatorial cage at 298 K and isotope effect factors.

r_{eq} / Å	r_{ax} / Å	$f_{complex}$	EIE	MMI	EXC	ZPE
3.0	2.04	69189	0.844	1.320	0.909	0.702
	2.525	32891	0.931	1.319	0.976	0.723
	3.0	24213	0.993	1.319	1.039	0.724
3.5	2.04	61558	0.948	1.322	0.888	0.806
	2.525	31643	0.967	1.321	0.931	0.786
	3.0	24366	0.987	1.321	0.980	0.762
3.75	2.04	59969	0.974	1.323	0.866	0.848
	2.525	31074	0.985	1.321	0.900	0.828
	3.0	24172	0.995	1.321	0.941	0.800
4.0	2.04	59233	0.986	1.323	0.846	0.878
	2.525	30790	0.994	1.322	0.873	0.861
	3.0	24046	1.000	1.322	0.904	0.837
∞	2.04	58381				
	2.525	30608				
	3.0	24052				

Notes on Table S10

All hydrogen atoms have mass m_H = 1. Because each symmetric axial structure [$H_2O…CH_3^+…OH_2$] is a TS both with and without the equatorial waters, each isotope effect for transfer into the cage is an EIE. However, the IPFR ($f_{complex}$) for each structure includes a quantum correction on the transition frequency for methyl transfer which (approximately) cancels in the ratio EIE = $f(\infty)$ / $f(r_{eq})$. The normal MMI factor is almost constant. For each value of r_{ax} the EXC becomes less inverse as r_{eq} decreases but the ZPE factor becomes more inverse and dominates the trend in the EIE. Some of these data are presented graphically in Figure 2 of the paper.

Table S11. B3LYP/aug-cc-pVDZ isotopic partition function ratios and factors for RS and TS, and KIEs, for methyl transfer within a constrained superheavy cage with r_{ax} = 2.04 Å.

		RS r_{eq} / Å				TS r_{eq} / Å				KIE r_{eq} / Å			
		3.0	3.5	3.75	4.0	3.0	3.5	3.75	4.0	3.0	3.5	3.75	4.0
D_3	IPFR	274385	23668	22907	22359	23617	20761	20183	19944	1.160	1.140	1.135	1.121
	QC									1.003	1.003	1.003	1.003
	MMI	0.336	0.332	0.331	0.331	0.347	0.342	0.342	0.342	0.969	0.969	0.968	0.969
	EXC	1.466	1.502	1.545	1.566	1.385	1.426	1.461	1.496	1.058	1.053	1.057	1.047
	ZPE	55581	47517	44835	43132	49343	42669	40561	39166	1.126	1.114	1.105	1.101
T_3	IPFR	4534493	3728891	3574577	3459265	3526567	2975164	2869461	2827638	1.286	1.253	1.246	1.223
	QC									1.006	1.006	1.006	1.006
	MMI	0.352	0.350	0.349	0.358	0.357	0.355	0.355	0.355	0.985	0.985	0.984	1.008
	EXC	2.010	2.101	2.196	2.234	1.836	1.933	2.009	2.082	1.095	1.087	1.093	1.073
	ZPE	6411169	5077315	4660873	4341584	5409525	4362471	4051919	3852361	1.185	1.164	1.150	1.127
^{13}C	IPFR	1.258	1.256	1.257	1.258	1.187	1.182	1.181	1.180	1.060	1.064	1.065	1.066
	QC									1.005	1.006	1.006	1.006
	MMI	1.000	1.000	1.000	1.000	1.000	1.000	1.000	1.000	1.000	1.000	1.000	1.000
	EXC	1.045	1.052	1.053	1.054	1.030	1.036	1.037	1.037	1.015	1.015	1.015	1.016
	ZPE	1.204	1.194	1.194	1.194	1.158	1.147	1.145	1.145	1.039	1.042	1.042	1.043
^{14}C	IPFR	1.545	1.540	1.542	1.540	1.383	1.371	1.369	1.368	1.117	1.123	1.126	1.126
	QC									1.009	1.010	1.011	1.011
	MMI	1.000	1.000	1.000	1.000	1.000	1.000	1.000	1.000	1.000	1.000	1.000	1.000
	EXC	1.091	1.104	1.107	1.105	1.060	1.072	1.074	1.074	1.029	1.029	1.030	1.028
	ZPE	1.416	1.395	1.393	1.393	1.317	1.292	1.288	1.288	1.075	1.080	1.081	1.082

Notes on Table S11.

Hydrogen atoms on "water" molecules have mass m_H = 999; hydrogen atoms on methyl group have m_H = 1.

Quantum corrections on transition frequencies are included in the IPFRs for TSs. The quantum corrections (QC) on the KIEs are all very close to unity, indicating the relative insignificance of tunneling in these model methyl transfer reactions. Some of these data are presented graphically in Figure 3 of the paper.
